# Ghost Beam Suppression in Deep Frequency Modulation Interferometry for Compact On-Axis Optical Heads

**DOI:** 10.3390/s21051708

**Published:** 2021-03-02

**Authors:** Oliver Gerberding, Katharina-Sophie Isleif

**Affiliations:** 1 Institute of Experimental Physics, University of Hamburg, Luruper Chaussee 149, D-22761 Hamburg, Germany; 2 Deutsches Elektronen-Synchrotron DESY, Notkestraße 85, D-22607 Hamburg, Germany

**Keywords:** laser interferometry, displacement sensing, ghost beams

## Abstract

We present a compact optical head design for wide-range and low noise displacement sensing using deep frequency modulation interferometry (DFMI). The on-axis beam topology is realised in a quasi-monolithic component and relies on cube beamsplitters and beam transmission through perpendicular surfaces to keep angular alignment constant when operating in air or in a vacuum, which leads to the generation of ghost beams that can limit the phase readout linearity. We investigated the coupling of these beams into the non-linear phase readout scheme of DFMI and implemented adjustments of the phase estimation algorithm to reduce this effect. This was done through a combination of balanced detection and the inherent orthogonality of beat signals with different relative time-delays in deep frequency modulation interferometry, which is a unique feature not available for heterodyne, quadrature or homodyne interferometry.

## 1. Introduction

Laser interferometric displacement sensing is a central tool in precision metrology, inertial sensing, quantum technologies and prominently, gravitational wave detection experiments [1]. Different flavours of such sensors are being studied, with dynamic range, sensitivity, bandwidth and linearity as major characteristics. Displacement sensors with multi-fringe dynamic and operational range and sensitivities below $10^{-12}\;m/\sqrt{\mathrm{Hz}}$ at frequencies from 100 Hz down to <1 mHz are being studied for future space-based instruments, such as gradiometers [2] and gravitational wave detectors [3], and for the readouts of low-frequency inertial sensors [4,5,6,7] and test masses in future ground-based gravitational wave detectors [8,9]. Deep frequency modulation interferometry (DFMI) is one of the techniques that is being developed and studied for these applications [10,11,12,13].

DFMI can be used to implement multiple displacement (and tilt) sensors with minimal complexity of the ultra stable optics, the so called optical heads. Its major feature is the multi-fringe dynamic range phase readout with single-beam, unequal-arm-length interferometer topologies, which are enabled by creating a deep phase-modulation-like interferogram from which the desired phase information can be extracted in real time [14]. An important second feature is the ability to reduce the effect of in-band laser frequency noise by using a fixed length, ultra-stable interferometer, or optical head, as a frequency reference to implement either an active stabilisation or a noise-subtraction algorithm [10,11,12]. The latter approach is made possible by a third feature, an additional readout of the macroscopic arm-length difference, or to be more precise, delay difference, from the interferogram that provides absolute ranging information.

**Figure 1:**
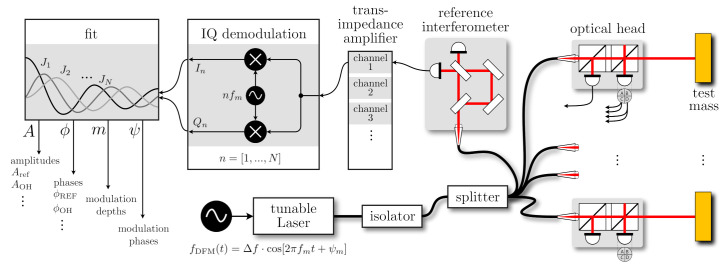
Conceptual schematic of multiple displacement and tilt sensors implemented using deep frequency modulation interferometry and the herein-discussed on-axis optical heads. The light from a laser, modulated sinusoidal in frequency, is split into fibres that provide light to several optical heads and a single, stable reference interferometer (here we depict an interferometer used in previous studies [12,15]). The currents from each photodiode or photodiode segment (when using quadrant detectors to enable differential wavefront sensing [16]) are converted into voltages by trans-impedance amplifiers, digitised and then fed into several demodulation pipelines that extract the complex amplitudes at *N* harmonics of the modulation frequency $f_{m}$ [17,18]. Afterwards, these complex amplitudes are fed into a fit algorithm to extract four parameters that should fully describe the interferogram.

A schematic of a DFMI multi-sensor displacement and tilt readout system is shown in Figure 1. Each optical head variant has an unequal arm length Mach–Zehnder or Michelson interferometer topology with one of the nominal beams $E_{S}$ propagating through a stable, short arm and another beam $E_{L}$ propagating towards the object of interest, or test mass, through a long arm. After beam recombination, the optical power sensed on the two complementary photodetectors can be approximated as

(1) $$P^{\pm }\propto {\left(E_{S}+E_{L}\right)}^{2}=\frac{E_{S}^{2}}{2}+\frac{E_{L}^{2}}{2}\pm E_{L}E_{S}\cos\left(\phi +m\cos\left(\omega_{m}t+\psi \right)\right).$$

The modulation index m=2πΔfτ depends on the differential delay between long and short arm τ and on the depth of the frequency modulation Δf. The interferometric phase encodes distance changes δL in the long arm ϕ=2δL·(2π/λ) via the laser wavelength λ. The underlying approximations are that the photodetector acts as a low-pass for the sum-frequencies and that the differential delay τ is much smaller than the period $T_{m}$ of the modulation frequency. Typical values are a modulation frequency of $f_{m}=1$ kHz, a frequency modulation depth of Δf=3.3GHz and an arm-length delay of $\tau =0.1\;m/c=0.\bar{3}\;$ns, leading to an effective modulation depth of m≈7. To implement the phase extraction the nominal signal from Equation (1) is rewritten using the Bessel functions of the first kind:

(2) $$\begin{array}{c} P^{\pm }\propto \frac{E_{S}^{2}}{2}+\frac{E_{L}^{2}}{2}\pm E_{L}E_{S}\left[J_{0}\left(m\right)\cos\left(\phi \right)+2\sum_{n=1}^{\infty }J_{n}\left(m\right)\cos\left(\phi +n\frac{\pi }{2}\right)\cos\left(n\left(\omega_{m}t+\psi \right)\right)\right] \end{array}$$

As first demonstrated by Heinzel et al. [14], one can now decompose the measured signal into a corresponding set of complex amplitudes using a discrete Fourier transform, or an IQ-demodulation, and then apply a Levenberg–Marquardt fit to extract the desired modulation parameters. The fit function is

(3) $$\begin{array}{c} P_{\mathrm{fit}}\left(n\right)=P_{f}J_{n}\left(m_{f}\right)\cos\left[\phi_{f}+n\frac{\pi }{2}\right]\left(\cos\left(n\psi_{f}\right)-i\sin\left(n\psi_{f}\right)\right)\;\mathrm{for}\;n=N_{l},\cdots ,N_{h} \end{array}$$

with $P_{f}$, $m_{f}$, $\phi_{f}$ and $\psi_{f}$ being estimated parameters and $N_{l}$ and $N_{h}$ being lowest and highest harmonics used for the analysis. Heinzel et al. found that for $N_{h}=10$, one can achieve reasonable small residuals when operating with modulation index values between m=6 and 9, and this approach has been used in various experimental demonstrations since. Real-time implementations of both the IQ demodulation and the fit algorithm have so far been demonstrated with estimation rates of up to 100 Hz [12,17]. Non-linearities in the phase readout were found to be caused by: (1) amplitude noise, which can be partly suppressed using fast feedback stabilisation, (2) non-linear laser frequency modulation in the laser [11], (3) amplitude and frequency dependence of the photodiode current detection chain, which can be accounted for by compensating the corresponding transfer function [14]. Active stabilisation of the modulation phase to ψ≈0rad has also been implemented and can reduce fit complexity by excluding the complex share of the harmonic amplitudes.

Related techniques using a similar laser modulation scheme with different readout algorithms have also been developed, one prominently with an additional, range-resolved multiplexing capability that is enabled by using stronger modulation indices and windowed signal extraction [19]. Multiplexing is realised with strong laser frequency modulations because the resulting beat signals become orthogonal for sufficiently large relative delay, a similar principle to the pseudo-random noise phase modulations that are used in digitally enhanced interferometer techniques to artificially reduce the coherence length [20].

DFMI is capable of performing displacement sensing with sub-picometer-level displacement noise using small, single-component interferometers as optical heads [12], and configurations that incorporate the reference interferometer into the same optic are also being studied [13]. The use of single-component, off-axis optical heads in previous and ongoing studies is in general motivated by the following advantages:

- Avoidance of ghost beams generated at points of transmission between inner and outer medium.
- Avoidance of back-reflections into the fibre and resulting parasitic beams.
- Minimal assembly (bonding) efforts.
- Avoidance of polarisation optics and/or major optical power losses.

The presence of ghost beams is well known to cause non-linear, cyclic phase noise in heterodyne and homodyne interferometers, and this especially includes parasitic reflections from fibres, which often have significantly higher phase dynamics than ghost beams generated within the often ultra-stable optical heads [21]. Reducing assembly efforts is also crucial when aiming for optimal performance at very low readout frequencies, which often implies the use of quasi-monolithic mounting structures. Off-axis designs can also be associated with the following disadvantages:

- Limited longitudinal operating range due to an inherent lateral beam-shift.
- Angular alignment change between air and vacuum operation, leading to increased alignment efforts with required pointing prediction.
- Angular coupling of refractive index fluctuations when operated in air.

The limited longitudinal range of operation is not a major factor for applications with very limited macroscopic length changes, such as test mass readouts in satellite gradiometers [22], but it can be limiting in applications where objects move more than several millimetres, and hence the absolute ranging information is of higher interest. The change of beam angles, while deterministic, can greatly influence adjustment strategies if one aims to operate the interferometers at optimal working points. For the prism-based design, the angular deviation between air and a vacuum was in the order of 1 mrad [12]. We consider manufacturing tolerances to be neither an advantage or disadvantage for either off or on-axis designs and argue that the associated effort is greatly dependent on each individual design and the underlying requirements.

To avoid some of the off-axis design disadvantages, we study here an on-axis design of a compact, quasi-monolithic, single-component optical head. In the following section we present the conceptual design of the optical head; discuss beam propagation, manufacturability and thermal effects; and show the simulated dependence of the interferometer signals on different test mass alignments. In Section 3 we then focus on the parasitic ghost beams that are unavoidable in our design and analyse how the DFMI readout algorithm can be adapted to reduce their influence on readout linearity by taking into account the orthogonality of beat signals with different effective modulation depths.

## 2. On-Axis Optical Head Design

**Figure 2:**
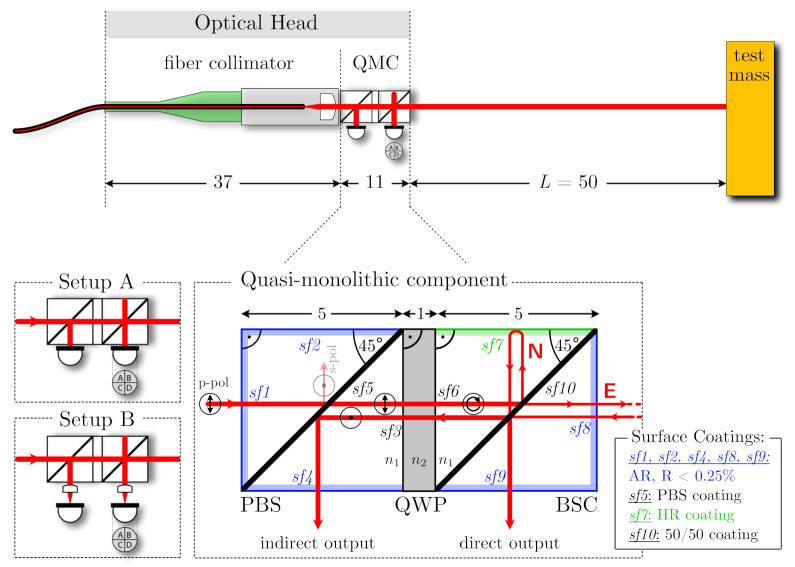
Sketch of the proposed on-axis optical head design. The core of the design is a quasi-monolithic component (QMC), which consists of a polarisation beam splitter (PBS) cube, a quarter waveplate (QWP) and a beam splitting cube (BSC) with 50% reflectivity at the diagonal surface. Nominal dimensions are given in units of 1 mm. The two setups A and B on the lower left indicate two variants for light detection on photodiodes (PDs) (simulations described below), one (A) with diodes mounted directly onto the component and the other one (B) with distant photodiodes and focusing lenses in between. The upper sketch shows the overall dimensions of the optical head when a fibre collimator is included. The QWP glass parts can be assembled using silicate bonding or optical adhesives after applying the specified coatings.

A top view schematic of the on-axis optical head design is shown in Figure 2. The core is a quasi-monolithihc component (QMC) in which two cube beamsplitters are joined via a quarter waveplate. The incoming beam from a fibre collimator enters the component from the left through surface 1 with p-polarisation and is transmitted through the polarisation selective coating at surface 5. From there the nominal beam propagates through the waveplate, which is aligned such that the beam entering the second cube has a circular polarisation. The next coating on surface 10 with a reflectance of 50% then creates an unequal arm-length Michelson interferometer with the short north arm, N, and the long east arm, E. Beam N is reflected by a highly reflective coating on surface 7 and beam E is reflected by the test mass, or object under testing. Both beams recombine at surface 10, and the two resulting interfered beams leave either directly through surface 9 or via a second transmission through the waveplate and then arrive at surface 4. The outer length of each cube is 5 mm and we assume a waveplate thickness of 1 mm (this might include an additional optical window for mechanical stability). All outer surfaces of the cubes are also coated with an anti-reflective coating to minimise the power of ghost beams.

The usage of polarisation elements has been studied for the Laser Interferometer Space Antenna (LISA) optical bench and test mass readout [23], and compact quadrature interferometers also rely on using different polarisation states and cube beamsplitters to decode the interferometric phase [5]. Other interferometer studies for LISA have also shown that it is beneficial to clean the polarisation delivered into displacement interferometers [24]. In the presented topology, the polarising surface and the waveplate have three nominal functions. (1) Any residual s-polarisation from the input beam is filtered out. (2) The indirect interference beam is not reflected back into the fibre and can be probed. (3) Reflections of the indirect interference beam at surfaces 3 and 4 cannot interfere at surface 10, because they have an orthogonal circular polarisation. Leakage of polarisation will be caused by either the waveplate, due to imperfect rotational alignment and deviations from its nominal thickness, or mismatches in reflectivity at surface 10 between s and p polarisation components.

We assume that the minimal distance *L* to the test mass is 5 cm. The paths of the two nominal beams are largely in common within the cube, with the propagation of the long arm towards the test mass being the major difference between them. Temperature-induced thermal expansion and changes of refractive index will cancel out to a large degree for the component internal beam path, leaving only the long-arm length change due to thermal expansion of the component as first order coupling. This effect is of course dependent on the mounting strategy of the interferometer and on the expansion of the spacer that defines the distance to the test mass, two effects which can partly cancel each other if materials with suitable coefficients of thermal expansion are used. For a characteristic length of 5 mm and a coefficient of thermal expansion of $\alpha =5\times 10^{-6}$, the uncompensated temperature coupling is in the order of $2.5\times 10^{-8}\;m/K$.

The dimensions of the optical head are compatible with a beam-diameter of about 1 mm, taking into account chamfers and that coatings typically do not extend into the corners on each surface. Compared to previous off-axis designs this leads to further reductions of component volume and size, giving even more prominence to the fibre collimator dimensions in the design of the optical head.

All parts that are used for constructing the QMC are standard optical components that can be manufactured, and are available, with low angular tolerances [25,26], providing, for example, sufficient perpendicularity and parallelism of the surfaces to enable two-dimensional interferometer assembly, as is done in the case of the LISA optical bench [27]. The dimensional tolerance of the components is largely uncritical for our design. Angular tolerances and matching the pathlength between surface 10 and surfaces 7 and 8 are the most critical to achieve minimal coupling of thermal and refractive index effects. Assembly of multi-component optical elements requires a bonding method, such as an optical adhesive or silicate bonding, and ultra-precise alignment [28,29]. The macroscopic alignment of the input beam and the resulting output beams is mainly driven by the need to avoid beam clipping, and with typical tolerances of the used components and an absolute beam positioning of about 100μm, this requirement should be fulfilled. The major fine adjustment needed is to ensure matching points of beam splitting and beam interference at the critical diagonal surface 5, which is the condition for maximum longitudinal range with a zero-angle of incidence on the test mass. This can be achieved by tuning the tip and tilt angles of the input beam and test mass, or of the optical head relative to the test mass as alternative to the latter. In the case of angular misalignment between the critical surfaces, the splitting and recombination point can still be matched, if input beam and test mass alignment (or again optical head orientation) are adjusted accordingly. Angular misalignment of surfaces transmitted by the input beam can be compensated in this process.

**Figure 3:**
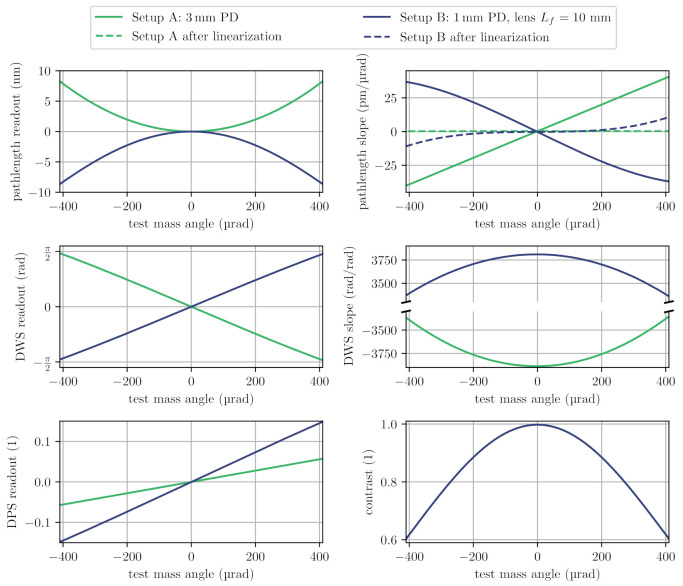
Simulated signals at the direct output ports for setups A and B (see Figure 2) under in-plane test mass rotation. The input beam parameters for both setups were 0.5 mm waist size radius and 15 mm waist position. The beam origin was 5 mm away from the PBS. The wavelength was 1550 nm. The distance between BSC and photodiode was 0.1 mm in Setup A. The distance to the lens was 0.1 mm in Setup B; there was a distance between the lens and photodiode of 9.0 mm.

We studied the influence of test mass tilts on the interferometer signals using the IfoCAD software library [30]. We assumed a Gaussian input beam with a waist diameter of 1.0 mm and a waist position at surface 5. We calculated signals for large (3 mm diameter) photodiodes that are mounted directly on the outer surfaces of the interferometer (setup A) and for smaller (1 mm diameter) photodiodes that are placed further away with some focusing lenses in between (setup B). Neglecting ghost beams, we derived interferometer contrast, tilt-to-length coupling and its slope. For a tilt-sensitive readout with quadrant photodiodes, we also computed the expected differential wavefront sensing, its derivative and the differential power sensing signals. We limited the analysis to a maximum tilt of about ±0.4 mrad to ensure beam clipping effects were negligible. The results of our simulation are shown in Figure 3.

Interferometric contrast within the analysed range of tilt stayed above 60% and we found a slightly angle-dependent differential wavefront sensing coupling factor of about 3750 rad/rad, comparable to the prism interferometer design [12]. The position of the focusing lens in setup B was fixed and not optimised further, and we found that the tilt-to-length coupling in this case was less linear. For both setups we also calculated the coupling after a linearisation with post-processing corrections that used the DWS readout for correcting the tilt-to-length coupling. Interestingly, this simple linearisation was more effective for setup A. These results show that the on-axis optical head has a comparable angle-dependency to similar compact interferometers, and that one can at least consider mounting the photodiodes directly next to, or onto the component to further reduce the footprint of the overall assembly.

## 3. Ghost Beam Analysis for Deep Frequency Modulation Interferometry

In an idealised set-up, with perfect alignment and anti-reflective coatings, all light sent into the optical head is used for the readout, no light is reflected back into the fibre, only the nominal polarisation is present and no parasitic ghost beams are generated. Since anti-reflective coatings and bonding interfaces have some residual reflection in any real device and polarisation leakage also occurs, we have to assume that non-negligible ghost beams are generated on various surfaces in our design. Theses parasitic beams will lead to additional interference signals that can limit the phase readout noise and linearity. Estimating the coupling of these parasitic beams to the phase readout is non-trivial for DFMI, because the effective modulation depth of each interference beat signal depends on the relative propagation delays between any two beams. This effect not only complicates the analysis; it also provides opportunities to adapt the phase estimation algorithm to reduce the influence of parasitic beams. For large differences in effective modulation depth, the resulting signals become orthogonal, a fact that is used in other interferometer techniques to implement multiplexing capabilities. In the following analysis we use this fact to reduce the influence of parasitic ghost beams that are caused by parasitic surface reflections.

Another technique we apply is prominently used in heterodyne interferometers to reduce the influence of ghost beams, the so-called balanced detection. It uses the two complementary output port signals of the interferometer, from which amplitude and phase are extracted and then combined in the complex plane to null the additional optical interference terms [21]. Application of this technique requires the phase readout to be sufficiently linear for the coupling of the respective ghost beam beat signals, which we analyse in the following.

Before we can begin the analysis, we must introduce some simplifications to make the equations more readable. In the following, we write interference signals without considering the constant DC terms ($P_{x}\propto E_{x}^{2}$); we set the units of the electric fields and of the resulting interference signals to 1 ($\left[P\right]=\left[E_{x}^{2}\right]=1$); we set the values of the two nominal beams in the north and east arms to unity ($E_{N}=E_{E}=1$); and we assume that all parasitic reflections are smaller than 0.25% ($r_{p}\leq 0.05$), leading to negligible degradation of the nominal beam amplitude ($t_{p}\approx 1$). We also include the assumption that the reflectivity of surface 10 is $r_{10}=1/\sqrt{2}$, which corresponds to an input power of $P_{\mathrm{in}}=2$. For ghost beam analysis, only the relative beam amplitudes are important, and we neglect other noise sources such as shot noise, readout noise and digitisation noise.

To deduce the DFMI signals, we assume the typical approximations when calculating the beat signals. We set the propagation delay of the short north arm at the point of interference and its phase to zero ($\tau_{N}=0\;s$, $\phi_{N}=0\;\mathrm{rad}$) and also set the modulation phase to zero (ψ=0rad). Finally, we use $c\left(t\right)=\cos\left(\omega_{m}t\right)$. In this notation the nominal beat signal for the two complementary output ports becomes

(4) $$P_{N,E}^{\pm }=\pm E_{N}E_{E}\cos\left[\phi_{E}+m_{E}\cos\left(\omega_{m}t\right)\right]=\pm 1\cos\left[\phi_{E}+m_{E}c\left(t\right)\right],$$

with a modulation depth $m_{E}=2\pi \Delta f\tau_{E}=2\pi \Delta f\left(\tau_{L}+\tau_{1}\right)\approx 2\pi \Delta f\left(2L\right)/c$ and the desired interferometric phase $\phi_{E}=\phi_{L}+\phi_{1}=2\delta L/\lambda \cdot 2\pi +\phi_{1}$. Here *c* is the speed of light, λ is the laser wavelength and $\phi_{1}$ is an additional, almost constant phase that accounts for slight differences $\tau_{1}$ between both arms within the cube. Both beams are interfered at surface 10 the associated phase shifts leads to the opposite sign for each output port, which is the basis for the above-mentioned balanced detection scheme. We assume a minimal distance to the test mass of L≥5cm.

**Figure 4:**
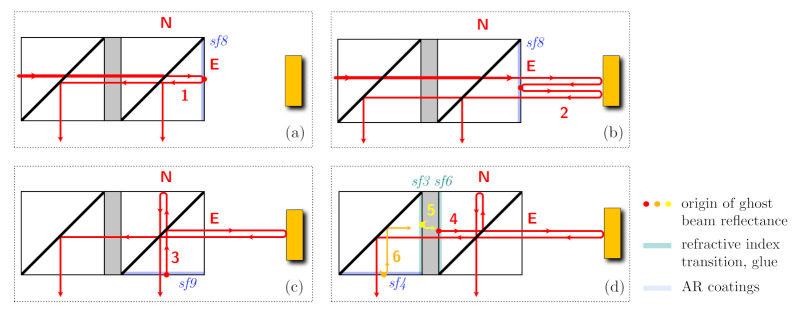
Propagation of individual, parasitic ghost beams generated by zero-angle transmission at internal and external surfaces of the QMC. (**a**) shows the path of beam 1, reflected directly at surface 8. (**b**) shows the path of beam 2, reflected at surface 8 after one trip through the long arm. (**c**) shows the path of beam 3, generated via a reflection of the interfered beams at surface 9 in the direct output. (**d**) shows the path of beams 4, 5 and 6, created by reflections in the indirect output path. Reflected Beams 1–4 lead to parasitic beat signals with the highest amplitudes, while beams 5 and 6 are somewhat suppressed by the polarisation optics.

Now we include the first parasitic reflection into our analysis, the internal reflection at surface 8, as shown in inset (a) of Figure 4. It generates an additional beam $E_{1}$ with an amplitude $|E_{1}|=0.05$, a delay that is very similar to the one in the north arm, $\tau_{1}\approx 0$, and a random phase $\phi_{1}$ that varies only very slowly and with negligible dynamics (due to the low coefficient of thermal expansion and assumed temperature stability). The resulting output power now contains three effective beat signals ($m_{E}=m_{L}+m_{1}$) and $\phi_{E}=\phi_{L}+\phi_{1}$:

(5) $$\begin{array}{cc} P_{N,E,1}^{\pm }=\pm & E_{N}E_{E}\cos\left[\phi_{L}+\phi_{1}+m_{E}c\left(t\right)\right] \end{array}$$

(6) $$\begin{array}{cc} \pm & E_{N}E_{1}\cos\left[\phi_{1}+m_{1}c\left(t\right)\right] \end{array}$$

(7) $$\begin{array}{cc} + & E_{E}E_{1}\cos\left[\phi_{L}+\left(m_{E}-m_{1}\right)c\left(t\right)\right] \end{array}$$

(8) $$\begin{array}{cc} =\pm & 1\cos\left[\phi_{E}+m_{E}c\left(t\right)\right] \end{array}$$

(9) $$\begin{array}{cc} \pm & 0.05\cos\left[\phi_{1}+m_{1}c\left(t\right)\right] \end{array}$$

(10) $$\begin{array}{cc} + & 0.05\cos\left[\phi_{E}-\phi_{1}+\left(m_{E}-m_{1}\right)c\left(t\right)\right] \end{array}$$

Please note that this result is not physical; it defies energy conservation because of the unitary transmission for the nominal beams. However, this description is sufficient to demonstrate our ability to effectively cancel all of the here listed parasitic contributions.

To probe into the effect of this signal on the phase estimation, we expand the three beat notes using the Bessel functions of the first kind and then we add their contributions to the injected harmonic amplitudes of the modulation frequency for each output port in a numerical simulation. Then we use a non-linear Levenberg–Marquardt fit operating on the harmonics $N_{l}$ to $N_{h}$ to estimate two sets of the three desired signal parameters, the amplitude $P_{f}$, the modulation depth $m_{f}$ and the interferometric phase $\phi_{f}$. The fit function we use is

(11) $$\begin{array}{c} P_{\mathrm{fit}}\left(n\right)=P_{f}J_{n}\left(m_{f}\right)\cos\left[\phi_{f}+n\frac{\pi }{2}\right]\;\mathrm{for}\;n=N_{l}\cdots N_{h} \end{array}$$

We also make the following assumptions: The pathlength difference corresponding to $\tau_{1}$ is smaller than 100μm, from which we estimate that $m_{1}=\tau_{1}/\tau_{L}\cdot m_{L}\leq 0.001\cdot m_{L}$ and with $m_{L}\approx 7$ this leads to $m_{1}\leq 0.007$.

**Figure 5:**
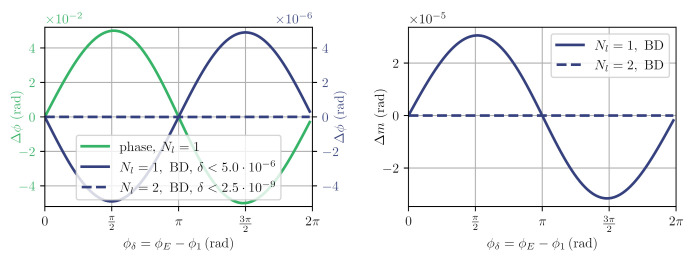
Simulated worst-case deviations from the nominal and estimated phases Δϕ; and the modulation index deviations, Δm, for signals according to $P_{N,E,1}^{\pm }$ and fit $P_{\mathrm{fit}}$ for $m_{L}=7.0,\phi =\pi /4$. The error reduction due to balanced detection (BD) and exclusion of the first harmonic are clearly visible.

Using $N_{l}=1$ and $N_{h}=10$, we find the expected non-linear behaviour of the difference between the two estimated and injected phases that depends on the phase difference $\phi_{\delta }=\phi_{1}-\phi_{L}$ and the absolute value of $\phi_{1}$. The strongest phase estimation error $\Delta \phi =\phi_{E}-\phi_{f}$ is shown as a green line in Figure 5. We derive the maximum strength of the unit-less non-linear coupling as

(12) $$\delta =\left|\frac{\Delta \phi }{\phi_{\delta }}\right|.$$

Since we assume that $\phi_{1}$ is a slowly changing quantity, largely driven by temperature effects, its phase dynamics will be below our desired phase noise levels in the order of $1\;\mu \mathrm{rad}/\sqrt{\mathrm{Hz}}$ and this means that we can evaluate the effect of the ghost beams on our readout purely with regard to the phase dynamics of the actual phase $\phi_{L}$, which is the test mass motion in combination with the average laser frequency variation, though the latter can be stabilised also to negligible values. This is true for the analysis of all other ghost beams discussed below as well, and we argue that ghost beams occurring in our optical head are fully negligible for the readout of phase variations in applications with very low signal dynamics. To improve on the non-linearity of δ<0.05, we then apply a balanced detection correction by computing the vector difference between the two output signals, effectively negating the influence of the $E_{E}E_{1}$ beat. Results of this approach are shown as solid blue line in Figure 5, reducing the non-linear coupling to $\delta <5\times 10^{-6}$. Errors in modulation depth estimation are plotted as well with $\Delta m=m_{f}-m_{E}$. The additional correction only suppresses the $E_{N}E_{1}$ beat, because it has the same sign in both output ports. Finally, we recognise that the modulation depth of $m_{1}$ is very small and that the $E_{E}E_{1}$ contributions are largely contained in the first harmonic N=1. Since this one is often contaminated by amplitude noise due to the laser modulation, we adapted the fit algorithm to operate only on the amplitudes of $N_{l}=2$ to $N_{h}=10$ and achieve the dashed, blue line results in Figure 5. This principle also works for other values of $m_{L}$, but at a value of $m_{L}=7$ we expect a minimal reduction in signal-to-noise ratio, because $J_{0}\left(m=7\right)\approx 0$, meaning the fit will ideally not evaluate this harmonic amplitude anyhow. As one can see from the right side of Figure 5, the estimation of the modulation index also significantly improves when excluding the first harmonic.

Thus far we have shown the use of balanced detection as a dedicated post-processing step, but we recognise that the separate fitting of two complementary output ports could be integrated into a single fit routine that also takes the presence of a parasitic ghost beam beat-note at the same modulation depth into account. Since the fit is inherently non-linear, we also hoped to increase robustness when making the fit function more physical for later experiments. We adapted our fit to operate on two sets of harmonics, with the indirect output port harmonics having a negative sign in their index, *n*, and we extend the fit function with two parameters, a ghost beam phase $\phi_{f,g}$ and amplitude $P_{f,g}$,

(13) $$\begin{array}{c} P_{\mathrm{fit},\mathrm{BD}}\left(n\right)=J_{\left|n\right|}\left(m_{f}\right)\left[\mathrm{sgn}\left(n\right)P_{f}\cos\left(\phi_{f}+\left|n\right|\frac{\pi }{2}\right)+P_{f,g}\cos\left(\phi_{f,g}+\left|n\right|\frac{\pi }{2}\right)\right] \end{array}$$

(14) $$\begin{array}{c} \mathrm{for}\;n=-N_{h},\cdots -N_{l},N_{l},\cdots N_{h} \end{array}$$

The fit with included balanced detection achieved the same linearity results in our simulation; it increased computation times by up to a factor of two and reduced the parameter error standard deviations by a factor of $\sqrt{2}$, which indicated a higher robustness.

In the next step of our analysis, we include the second parasitic reflection from surface 8, as shown in inset (b) of Figure 4. This reflection occurs when the long arm beam is transmitted back into the component, creating a second round-trip beam $E_{2}$ with an amplitude $|E_{2}|=0.05$, a delay that is twice the one of the long arm, $\tau_{2}\approx 2\cdot \tau_{L}+\tau_{1}$, and a phase $\phi_{2}=2\cdot \phi_{L}+\phi_{1}$. When we now calculate the resulting output powers we have a total of six beat signals ($\phi_{L}=\phi_{E}-\phi_{1}$):

(15) $$\begin{array}{cc} P_{N,E,1,2}^{\pm }=\pm & E_{N}E_{E}\cos\left[\phi_{E}+m_{E}c\left(t\right)\right] \end{array}$$

(16) $$\begin{array}{cc} \pm & E_{N}E_{1}\cos\left[\phi_{1}+m_{1}c\left(t\right)\right] \end{array}$$

(17) $$\begin{array}{cc} \pm & E_{N}E_{2}\cos\left[2\phi_{L}+\phi_{1}+\left(2m_{L}+m_{1}\right)c\left(t\right)\right] \end{array}$$

(18) $$\begin{array}{cc} + & E_{E}E_{1}\cos\left[\phi_{L}+m_{L}c\left(t\right)\right] \end{array}$$

(19) $$\begin{array}{cc} + & E_{E}E_{2}\cos\left[\phi_{L}+m_{L}c\left(t\right)\right] \end{array}$$

(20) $$\begin{array}{cc} + & E_{1}E_{2}\cos\left[2\phi_{L}+2m_{L}c\left(t\right)\right] \end{array}$$

(21) $$\begin{array}{cc} =\pm & 1\cos\left[\phi_{E}+m_{E}c\left(t\right)\right] \end{array}$$

(22) $$\begin{array}{cc} \pm & 0.05\cos\left[\phi_{1}+m_{1}c\left(t\right)\right] \end{array}$$

(23) $$\begin{array}{cc} \pm & 0.05\cos\left[2\phi_{E}-\phi_{1}+\left(2m_{L}+m_{1}\right)c\left(t\right)\right] \end{array}$$

(24) $$\begin{array}{cc} + & 0.05\cos\left[\phi_{E}-\phi_{1}+m_{L}c\left(t\right)\right] \end{array}$$

(25) $$\begin{array}{cc} + & 0.05\cos\left[\phi_{E}-\phi_{1}+m_{L}c\left(t\right)\right] \end{array}$$

(26) $$\begin{array}{cc} + & 0.0025\cos\left[2\phi_{E}-2\phi_{1}+2m_{L}c\left(t\right)\right] \end{array}$$

**Figure 6:**
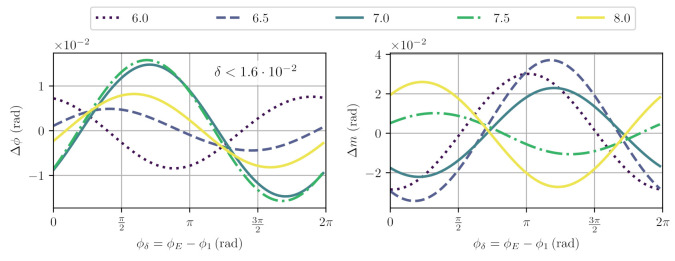
Simulated, worst-case deviations from nominal and estimated phase and modulation indices for signals according to $P_{N,E,1,2}^{\pm }$, and fit $P_{\mathrm{fit},\mathrm{BD}}$ for different values of modulation index $m_{L}$. The maximal non-linearity was found for $m_{L}=7.5$ with $\delta <1.6\times 10^{-2}$.

The second beat has already successfully been suppressed by excluding the first harmonic, $N_{l}=1$, from the fit. Beat signals four and five can again be suppressed using balanced detection. The third and sixth beat signals, however, cannot be canceled with the previous fit, but the sixth beat is already significantly smaller in amplitude. We analysed the phase and modulation index error when probing $P_{N,E,1,2}^{\pm }$ with the fit function $P_{\mathrm{fit},\mathrm{BD}}$, and the results are shown in the upper plots in Figure 6 for different values of *m* and for the worst-case value of $\phi_{1}$. The non-linearity is below $\delta <1.6\times 10^{-2}$, already an improvement against the nominal beat amplitude of 0.05 due to the higher modulation index of this disturbance.

Since the third beat signal has twice the effective modulation depth, we can use the orthogonality to expand our fit algorithm such, that it searches for two additional parameters, the power of the second tone $P_{f,2m}$ and its phase $\phi_{f,2m}$. Now we are estimating a total of seven parameters, and the fit function is

(27) $$\begin{array}{c} P_{\mathrm{fit},\mathrm{BD},2m}\left(n\right)=J_{\left|n\right|}\left(m_{f}\right)\left[\mathrm{sgn}\left(n\right)P_{f}\cos\left(\phi_{f}+\left|n\right|\frac{\pi }{2}\right)+P_{f,g}\cos\left(\phi_{f,g}+\left|n\right|\frac{\pi }{2}\right)\right] \end{array}$$

(28) $$\begin{array}{c} +\mathrm{sgn}\left(n\right)P_{f,2m}J_{\left|n\right|}\left(2m_{f}\right)\cos\left[\phi_{f,2m}+\left|n\right|\frac{\pi }{2}\right] \end{array}$$

(29) $$\begin{array}{c} \mathrm{for}\;n=-N_{h},\cdots -N_{l},N_{l},\cdots N_{h}. \end{array}$$

**Figure 7:**
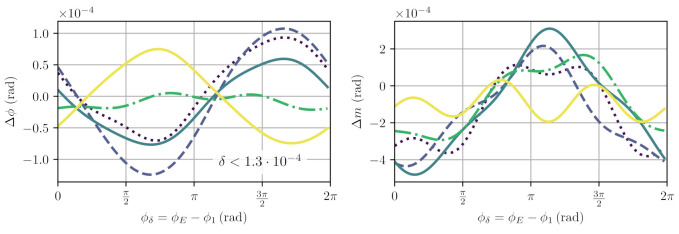
Simulated, worst-case deviations from nominal and estimated phase and modulation indices for signals according to $P_{N,E,1,2}^{\pm }$, and fit $P_{\mathrm{fit},\mathrm{BD},2m}$ m for different values of modulation index $m_{L}$. The maximal non-linearity was found for $m_{L}=7.5$ with $\delta <1.3\times 10^{-4}$. Error and non-linearity for phase and modulation index improved by more than two orders of magnitude in comparison to results shown in Figure 6.

The results using this fit are shown in Figure 7 for different values of *m* and for the worst-case value of $\phi_{1}$. With the inclusion of the second harmonic fit, we reduce the phase readout linearity to $\delta <1.3\times 10^{-4}$ and the error in modulation depth estimation to $|\Delta m|<3\times 10^{-4}$. The remaining non-linearities are understood to be dominated by the sixth beat signal. However, its effect is not only suppressed by its smaller amplitude, but also by its inherent orthogonality. A further extension of the fit to also account for the last beat is of course possible, but we do not explore this here.

Thus far we have shown that we can adapt the DFMI readout algorithm to account for parasitic reflections occurring at surface 8 of our design in such a way that non-linearity can be reduced to levels below 0.1%. We have not studied the influence of a possible third round-trip signal, but expect it to be even less relevant, due to its even smaller amplitude and higher delay. Handling ghost beams generated at surface 8 under zero-degree transmission is the most crucial aspect to achieving our goal of an on-axis design with no alignment change between air and vacuum, and further experimental exploration of linearity will first have to deal with other non-linearities from, for example, the laser frequency modulation. A maximum error $\Delta m=5\times 10^{-4}$ for the extracted modulation depth corresponds to an error in absolute ranging of less than 4μm for $m_{L}=7$ and L=5cm, excluding additional errors due to calibration and noise.

**Figure 8:**
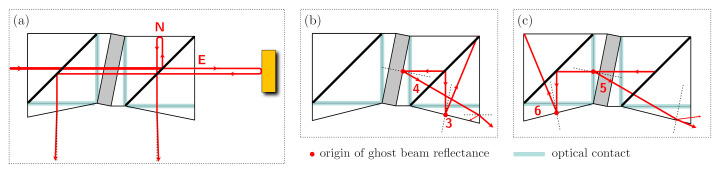
Shown are sketches of an alternative QMC design that employs intentional wedge angles to reduce the influence of ghost beams 3, 4, 5 and 6, as depicted in Figure 4. The wedges can either be realised by machining more complex shapes than right angle prisms or by including additional wedged windows with matching refractive indices that are bonded at the marked surfaces with very low transmissivity contacts. (**a**) shows the propagation of the nominal beams. (**b**) shows the deflection achieved for ghost beams 3 and 4. (**c**) shows the deviation achieved for ghost beams 5 and 6. An optimised design would need to take the optimal birefringence of the waveplate and the changing refractive indices into account.

As shown in the two lower sketches of Figure 4, the design is sensitive to additional ghost beams generated at other surfaces. The reflections 3 and 4 especially can lead to relevant non-linearities, as discussed below. Reflections 5 and 6 are to some degree suppressed, because they would enter the right cube with an orthogonal circular polarisation. All of these zero-degree reflections not happening at surface 8 are not required to enable an on-axis design and could be suppressed with additional modifications and more complex topologies of the quasi-monolithic component. A variant that retains the on-axis design but is specifically adjusted to reduce the influence of reflections 3, 4, 5 and 6 is sketched in Figure 8. The parasitic beams have an intentional angle deviation and a significantly reduced interferometric beat signal amplitude, should they end up on the photodetector.

To complete the analysis for the design based on perpendicular external surfaces, we analyse the effect of ghost beam 3 (beam 4 would behave very similar, but its power depends on the refractive index mismatch and the bonding technique). To simplify the description, we assume from here on that the differential delay is negligible $\tau_{1}\approx 0$, we define a characteristic optical path length $d=2\times 5\;\mathrm{mm}\cdot n_{glass}\approx 15\;\mathrm{mm}$ for beams that bounce through the cube once and we discard any secondary ghost beams. If the already interfered signal is reflected back into the interferometer, we have to add a total of four additional beams to our analysis. We name these beams according to their travel paths; for example, the beam $E_{\mathrm{NN}}^{3}$ is the original beam $E_{N}$, reflected at surface 9, then split at surface 10 and reflected back at surface 7 to the interference point. The effective delay for this beam in our notation is $\tau_{\mathrm{NN}}=\tau_{N}+\tau_{d}=0+d/c$, with *c* being the speed of light, and the amplitude is $|E_{\mathrm{NN}}^{3}\left|=\right|E_{N}|\cdot r/2=0.025$. The interference between all eight relevant beams will consist of 28 beat signals. The matrix in Table 1 lists the effective modulation depth for each beat signal and the values below the diagonal include a negative sign if the beat has a phaseshift of π at the indirect output.

With the previous analysis we covered all signals of the upper left quadrant of this matrix. The lower right quadrant produces beat signals at the same modulation depth; as covered by our fit algorithm, their phases should by largely coherent and the corresponding beat amplitudes are in the order of 0.1%. Critical additions are the beat signals in light gray that contain a delay corresponding to the characteristic length *d*, which is $m_{d}=m_{L}\cdot d/\left(2L\right)\approx 0.15m_{L}$ for a long arm length of L=5cm. For $m_{L}=7$ this gives $m_{d}=1.05$. Since the phase of each of these eight critical beat signals might have some additional, relatively constant phase contribution, we evaluated their impacts individually by adding only one of them add a time to $P_{S,L,1,2}^{\pm }$ and measuring the non-linearity for different additional phase values and values of $m_{L}=6$ to 8. The maximal δ determined for each beat signal is shown in Table 2, together with the effective modulation depth values for $m_{L}=7$.

**Figure 9:**
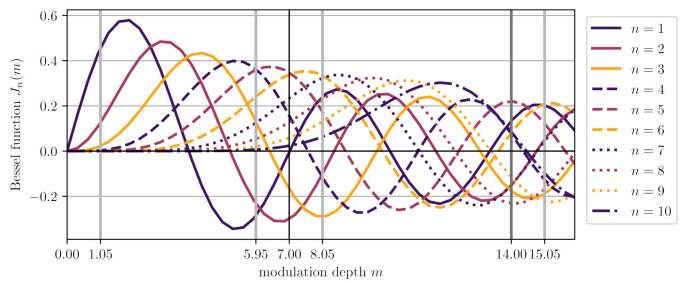
Shown are the Bessel functions of the first kind for the first 10 harmonics. The vertical lines indicate the modulation depth values that correspond to the major parasitic beat signals present when considering ghost beams 1, 2 and 3 for $m_{L}=7$.

As expected from our previous analysis, the beat signals further away in modulation depth contributed less, and the tones that were suppressible via balanced detection also had a slightly weaker influence when compared to other signals at the same modulation depth. Figure 9 shows the $N_{l}=1$ to $N_{h}=10$ Bessel functions of the first kind together with vertical lines indicating the modulation depth of the relevant beat signals. Luckily, even beat signals close to the nominal modulation depth ($\pm m_{d}$) were to some degree orthogonal. By adding the effect from each of these eight parasitic beat signals, we arrived at a conservative, total non-linearity of δ<0.0027 (for $m_{L}=6.5$, the worst case), which is roughly an order of magnitude below the amplitude of each of these beats of r/2=0.025.

Since the ratio of *d* and *L* can be adjusted, one could study other combinations that might be more or less susceptible to these couplings. Applying even more complex fit algorithms is of course also possible, but the number of extracted parameters (seven for $P_{\mathrm{fit},\mathrm{BD},2m}$) should generally not exceed the number of harmonics analyzed (18 for $P_{\mathrm{fit},\mathrm{BD},2m}$), so overall increases of modulation depth and harmonics analyzed might be required. Future iterations and extension of the fit could also make use of more correlations within the signal—for example, fixed amplitude and phase relations—to effectively reduce the number of fit parameters.

Estimating the coupling of ghost beams that are caused by polarisation leakage requires a different analysis not covered here and will also depend strongly on the quality of the optics, coatings and alignment. The most dominant polarisation ghost beams will create beats at the nominal modulation depth, making them non-suppressible by extending the fit. Since such beats will follow the nominal phase, to first order their coupling is expected to be, at most, a second order effect. An experimental study of a similar combination of a polarising beamsplitter and a quarter waveplate for LISA found no measurable non-linearity within their sensitivity [23]. Comparing this to the QMC is, however, not straight-forward because the interferometer topology is more complex, with the Michelson beamsplitter (surface 10) as a crucial difference. Practically speaking, one can try to add additional optics to clean the output beams if polarisation leakage should become a dominating effect.

For actual implementations, we recommend operating the optical head first in a calibration mode in which the long arm beam is dumped. In this mode, only beat signals between $E_{N}$, $E_{1}$ and $E_{\mathrm{NN}}$ will be present, and one can, for example, increase the laser power to enable a higher signal-to-noise ratio measurement of the parasitic beats that are otherwise dominated by the main measurement beat and readout noise. From such a measurement, the modulation depth $m_{1}$, $m_{d}$ and the corresponding electric field amplitudes might be estimated with higher accuracy before operating with a test mass, and in turn, this also quickly reveals the quality of the anti-reflective coatings and might indicate whether other parasitic beats are present.

The noise sources neglected in the above analysis will always limit the displacement readout noise floor to similar levels as in previous implementations, if the same opto-electronics and power levels are used, to about $230\;\mathrm{fm}/\sqrt{\mathrm{Hz}}$ [12]. Without expanding the fit algorithm, non-linear noise is created in our design for large signal dynamics that are dominated by $E_{1}$ and $E_{2}$ and scales linearly with the square root of the dominating parasitic reflectivity *R*. For R≤0.25% the extended fit algorithms bring the maximum non-linearity caused by ghost beams down to a level of $\delta <3\times 10^{-3}$, which is slightly below the current experimentally demonstrated level of $\delta \approx 1\times 10^{-2}$ achieved with an off-axis topology [12]. Larger values of *R* therefore reduce the phase measurement spurious-free dynamic range but might be acceptable if the test mass motion is sufficiently small during times in which the full phase readout sensitivity is required. Deviations of the beam splitter cube reflectivity from 50% will reduce the suppression of ghost beams provided by balanced detection, but small amplitude differences in both output ports can be accounted for in post-processing [21].

## 4. Conclusions and Outlook

Realising on-axis optical heads for DFMI is an interesting approach to enable picometer-level displacement and nano-radian tilt sensing and micrometer-accuracy absolute ranging that can be realised using compact QMCs. Major advantages are the extended longitudinal measurement range and fixed beam-angles, which will simplify alignment strategies, especially if several sensors are to be used to probe a single test mass. One expected disadvantage of our design, non-linearity due to zero-angle generation of ghost beams, can be suppressed by extending the readout algorithm of DFMI. Balanced detection and fitting of higher modulation depth signals were shown to be effective extensions that reduce ghost-beam-induced non-linearity in simulations to levels below 0.3% for a straightforward and below 0.01% for an extended design when several parasitic signals with a relative amplitude of ≈5% were present.

The complexity and orthogonality of DFMI beat signals also provides opportunities for other multiplexing and ghost beam suppression schemes, and reducing non-linearities due to the laser modulation [11]. The reduction in optical head size and its other benefits are paid for by increasing the phase extraction complexity and computational effort. The increase in computation time between the initial fit, $P_{\mathrm{fit}}$, and the final fit, $P_{\mathrm{fit},\mathrm{BD},2m}$, was less than four in our simulations. Laboratory tests of such sensors and real-time algorithm implementations will reveal the compatibility of such approaches for any given application; they will give insights into effects caused by polarisation leakage; and further improvements in the signal processing and phase estimation might compensate for the computational effort as well. It is worthwhile to conclude with the fact that any real DFMI interferogram contains a multitude of information, which makes optimal phase extraction an arithmetical and numerical challenge that is, however, rewarded with additional information that could not be extracted in homodyne or heterodyne interferometers.

## Figures and Tables

**Table 1:** Summary of all relevant beat signals when considering reflections 1, 2 and 3. Parasitic signals with an amplitude of *r* are highlighted in gray, signals with an amplitude of r/2 are highlighted in light gray and all other parasitic signals have an amplitude $\leq r^{2}$. Some signals are suppressed using balanced detection, because they have different signs in the direct (above the diagonal) and indirect (below the diagonal) output port, just like the nominal signal.

$m_{x}$	$E_{N}$	$E_{E}$	$E_{1}$	$E_{2}$	$E_{\mathrm{NN}}^{3}$	$E_{\mathrm{NE}}^{3}$	$E_{\mathrm{EN}}^{3}$	$E_{\mathrm{EE}}^{3}$
$E_{N}$		L	≈0	+2L	*d*	+(d+L)	d+L	+(d+2L)
$E_{E}$	−L		L	L	+(L−d)	*d*	+d	d+L
$E_{1}$	≈0	L		2L	+d	d+L	+(d+L)	d+2L
$E_{2}$	−2L	L	2L		+(2L−d)	L−d	+(L−d)	*d*
$E_{\mathrm{NN}}^{3}$	*d*	−(L−d)	−d	−(2L−d)		+L	L	+2L
$E_{\mathrm{NE}}^{3}$	−(d+L)	*d*	d+L	L−d	−L		≈0	L
$E_{\mathrm{EN}}^{3}$	d+L	−d	−(d+L)	−(L−d)	L	≈0		+L
$E_{\mathrm{EE}}^{3}$	−(d+2L)	d+L	d+2L	*d*	−2L	L	−L	

**Table 2:** Parasitic beat signals from reflection 3 with an amplitude of r/2; those with changing sign are not suppressed using balanced detection. The modulation depth of each beat is shown together with the maximum non-linearity factor extracted by applying the fit function $P_{\mathrm{fit},\mathrm{BD},2m}$.

*x*	$E_{\mathrm{NN}}^{3}$	$E_{\mathrm{NE}}^{3}$	$E_{\mathrm{EN}}^{3}$	$E_{\mathrm{EE}}^{3}$
$E_{N}$	*d*	±(d+L)	d+L	±(d+2L)
$E_{E}$	±(L−d)	*d*	±d	d+L
$m_{L}=7$	$E_{\mathrm{NN}}^{3}$	$E_{\mathrm{NE}}^{3}$	$E_{\mathrm{EN}}^{3}$	$E_{\mathrm{EE}}^{3}$
$E_{N}$	1.05	±8.05	8.05	±15.05
$E_{E}$	±5.95	1.05	±1.05	8.05
$\delta_{m_{L}=7}$	$E_{\mathrm{NN}}^{3}$	$E_{\mathrm{NE}}^{3}$	$E_{\mathrm{EN}}^{3}$	$E_{\mathrm{EE}}^{3}$
$E_{N}$	$3.6\times 10^{-5}$	$\pm 5.2\times 10^{-4}$	$3.4\times 10^{-4}$	$\pm 3.8\times 10^{-4}$
$E_{E}$	$\pm 5.0\times 10^{-4}$	$3.6\times 10^{-5}$	$\pm 5.6\times 10^{-5}$	$3.4\times 10^{-4}$
